# Older Adult Willingness to Use Fully Autonomous Vehicle (FAV) Ride Sharing

**DOI:** 10.3390/geriatrics6020047

**Published:** 2021-04-29

**Authors:** Alexa L. Siegfried, Alycia Bayne, Laurie F. Beck, Katherine Freund

**Affiliations:** 1 NORC at the University of Chicago, 4350 East–West Hwy, Suite 800, Bethesda, MD 20814, USA; Bayne-Alycia@norc.org; 2 Centers for Disease Control and Prevention (CDC), 4770 Buford Hwy NE, MS S106-9, Atlanta, GA 30341, USA; ldf8@cdc.gov; 3 ITNAmerica, 90 Bridge St, Suite 210, Westbrook, ME 04092, USA; Katherine.Freund@ITNAmerica.org

**Keywords:** ride share, fully autonomous vehicles, self-driving cars, transportation options, safety, mobility, aging

## Abstract

In the United States, older adults (age 65 and older) rely on private automobiles for transportation. For those who stop driving, access to alternative modes of transportation is important for health, wellbeing, mobility, and independence. This paper explores older adult willingness to use fully autonomous vehicle (FAV) ride sharing and the features or services of FAV ride sharing that would make them willing to take a ride. These data were gathered as part of a larger qualitative research study designed to explore the factors affecting older adult use of ride share services. For the larger study, we conducted 68 telephone interviews with older adults, and 10 in-person focus groups with 56 older adults, including individuals who both used and never used ride share services. We used a convenience sample recruited by study partners, including ride share and transportation services and a recruitment firm. The predominant thematic findings of the qualitative analysis included a desire for a proven safety record in terms of performance and technology, followed by dependability and accuracy of FAV ride sharing. Older adults’ concerns about FAV ride sharing included safety concerns and preferences for social interaction with drivers. Ride share services that use FAVs in the future may need to tailor transportation offerings for older adults to increase their willingness to use FAVS to support their mobility and social needs.

## 1. Introduction

Mobility is defined as “the ability to safely and reliably go where you want to go, when you want to go, and how you want to get there” [1]. In the United States (U.S.), mobility is characterized by a high reliance on the private automobile [2]. However, there is a decline in driving as individuals age; data show that the number of daily trips as a driver of a private automobile decreases with age, while the number of daily trips as a passenger in a private automobile increases [2]. Additionally, age-related changes in cognitive function, vision, and motor abilities may negatively affect the driving safety of older adults [3]. These types of changes in physical and mental health may contribute to driving cessation [4]. Driving cessation among older adults results in decreased mobility, which has negative effects on social connectedness, general health and wellbeing, and independence, among other impacts [4,5,6].

Alternative modes of transportation are important for ensuring the continued mobility of older adults who cease driving. An alternative mode of transportation is defined as a “mode of transportation other than driving one’s self in a private vehicle” [7]. For older adults who stop driving, alternative modes of transportation may include public transportation such as a bus or train, walking, paratransit services, taxis, and ride share services, including non-profit and for-profit services [8]. In 2018, nearly one-quarter (24%) of U.S. adults age 50 and older reported having ever used a ride share service, up from 7% in 2015 [9].

In recent years, for-profit ride share services, referred to as transportation network companies (TNCs), have used technology to connect riders with drivers on demand, using a mobile smartphone application. Current and future advances in technology will continue to impact available transportation options for older adults—one such being autonomous vehicles (AVs). Ongoing research, development, and testing have advanced AV technology, bringing fully autonomous vehicles (FAVs) closer to reality. There are six levels of driving automation, defined by the Society of Automotive Engineers, that range from no automation (Level 0) where the human driver does all of the driving to full automation (Level 5) where the vehicle performs all driving tasks under all conditions [10]. In Level 5 automation, the vehicle does not require human interaction or a driver to operate the vehicle [11,12]. While FAVs are not widely available to the general public, companies are testing them and advancing toward higher levels of automation. For example, Waymo, Google’s self-driving car project, has opened FAV rides to customers of its ride-hailing service, Waymo One, in Phoenix, Arizona [13]. In another example, Cruise, an AV company headquartered in San Francisco, California, and partnered with GM and Honda, received a permit from the California Division of Motor Vehicles to remove human backup drivers from its self-driving cars [14].

AV technology has the potential to profoundly impact transportation and mobility. The potential benefits of adopting AV technology include enhanced mobility, efficiency and convenience, economic and societal benefits, and safety [11,15]. AV technology also has the potential to delay driving retirement among older adults [6] and increase mobility among those who have stopped driving [16]. However, for older adults to realize the potential benefits of AVs, they need to understand and trust the technology [5,6]. Older adults who are familiar with self-driving cars reported higher perceived trust and perceived safety of the technology [17], and in an AV simulator study of older adults, early findings indicate that exposure to AV technology may increase trust and perceived safety of AV technology [5].

Additional research has explored older adult attitudes toward AVs. For example, an online survey of adults age 60 and older found positive acceptance of self-driving vehicles, described as “willingness to integrate self-driving vehicles in their travel habits if they currently cannot drive or become unable to drive in the future” [17]. Additionally, focus groups with older adults regarding how they imagined their future use of AVs revealed a strong preference for AVs that are available on demand [16]. Conversely, several studies have found that older adults are less likely than younger populations to be willing to ride in a self-driving car [18,19]. Despite the belief that self-driving vehicles may contribute to increased mobility and independence, which older adults in one study identified as potential benefits of AVs [20], older adults have voiced concerns about AV technology related to reliability, safety, and trust regarding self-driving cars [20,21].

As AV technology continues to evolve and emerge, there remains a need to better understand older adult attitudes towards and perceptions of FAVs, particularly regarding FAV ride sharing, and their willingness to use this emerging technology. To assess older adult perceptions of using FAVs in ride sharing, we analyzed qualitative data gathered through a study conducted to explore the barriers and facilitators of older adult use of ride share services. This paper provides new information regarding older adult willingness to use and attitudes towards FAV ride sharing.

## 2. Materials and Methods

The data presented in this paper were gathered as part of a larger qualitative study (Barriers and Facilitators of Older Adults’ Use of Ride Share Services Study) conducted by NORC at the University of Chicago, with funding from the Centers for Disease Control and Prevention (CDC), to study the attitudes and beliefs of older adults towards using ride share services [22]. Using a qualitative research design, NORC collected primary data through interviews and focus groups with adults aged 18 and older. Each respondent was asked the following question: “If there was a ride share service that used self-driving or “driverless” automobiles, what features or services would make you willing to take a ride?” Additionally, NORC asked each respondent to report on individual characteristics including demographic data (gender, age, race, ethnicity, and geographic location); prior use of ride share services; and if they held a current driver’s license. NORC gathered these data on individual characteristics by self-report for interview respondents and through written questionnaires for focus group respondents. Responses were obtained between April and October 2019. The present study focused on FAVs (level 5) and used the terms self-driving cars and FAVs synonymously.

Data from 124 older adults were included in this study. The data were gathered through semi-structured telephone interviews (*n* = 68) and in-person focus group discussions (*n* = 56) with older adults. We used a convenience sample recruited by study partners: one for-profit ride share service; one concierge service that schedules rides with TNCs; six non-profit ride share services; and a third-party recruitment firm. Study partners shared a flyer and information with their members via newsletters, telephone, and email. People interested in participating contacted NORC using a toll-free number and answered a set of screening questions to determine eligibility to participate in the study. Eligibility criteria included age (older adults were age 65 and older), ride share use (“users” were defined as people who had ever used a ride share service; “non-users” were defined as people who had never used a ride share service), and employment by a ride share service (those ever employed were not eligible). Telephone interview respondents were recruited from across the U.S. and focus group respondents were recruited from within one metropolitan location (Bergen County, NJ, USA) and one micropolitan location (Fayette County, KY, USA). The study was reviewed by the NORC Institutional Review Board (IRB00000967, Federal Wide Assurance #FWA00000142), and received approval from the Office of Management and Budget (OMB No. 0920-1154).

We reviewed, cleaned, and analyzed the data using MS Word, MS Excel, and NVivo (QSR International Pty Ltd., Melbourne, Australia). We conducted quantitative data analysis to generate frequencies for respondents’ characteristics, including age group, gender, geographic location, race, ethnicity, use of ride share services, and having a driver’s license. We also produced descriptive statistics of respondents’ willingness to use FAV ride sharing; we categorized qualitative responses as “yes”, “no”, or “unsure” and calculated frequencies in Excel. Those who did not provide a response included focus group respondents who were asked the question by the NORC moderator but did not contribute to the discussion, nor provide a response that could be categorized for analysis. We categorized these individuals as “no response” and included those frequencies in the data output. We conducted qualitative data analysis of all responses to identify key themes across the interviews and focus groups. Data from interviews and focus groups were combined to facilitate the identification of key themes during qualitative analysis, and because the same question regarding FAV ride sharing was asked verbatim of all participants. All qualitative data were coded in NVivo by a five-member coding team, using a codebook developed through code query, key word searches, or a combination. Interrater reliability (IRR) was measured using Cohen’s Kappa coefficient, and the team achieved almost perfect agreement, with an IRR of 0.82 [23]. Four key themes related to the desired features of FAV ride sharing that would make older adults willing to use FAV ride sharing emerged and sample quotes were identified. We extracted and quantified these data in MS Excel to calculate frequencies for desired features of FAV ride sharing. All findings were derived from the data and agreed upon by all authors.

## 3. Results

### 3.1. Characteristics of Respondents

Table 1 presents the characteristics of respondents. Among the 124 older adults who participated in the data collection, ages ranged from 65 to 99 years, with approximately half of participants aged 65 to 74 (52.5%, *n* = 62), one quarter aged 75 to 84 (27.1%, *n* = 32), and one-fifth aged 85 and older (20.3%, *n* = 24). More than two-thirds were female (70.2%, *n* = 87) and nearly one-third were male (29.8%, *n* = 37). Nearly two-thirds of respondents resided in a large metropolitan area (63.9%, *n* = 78) and about one-third resided in a small metropolitan area (36.1%, *n* = 44). The majority of respondents were white (97.5%, *n* = 116) and not Hispanic or Latino (98.4%, *n* = 121). Nearly three quarters of the respondents reported having a driver’s license (72.1%, *n* = 88).

### 3.2. Willingness to Use FAV Ride Sharing

Table 2 presents data on older adult willingness to use FAV ride sharing. More than half of the older adults (58.9%, *n* = 73) said they were not willing to use FAV ride sharing. One in five older adults (21.0%, *n* = 26) said they would be willing to use FAV ride sharing. The remaining respondents were either unsure about their willingness to use FAV ride sharing or did not respond to the question.

Some respondents explained the reasons they were not willing to use FAV ride sharing. Nearly half of the respondents who were not willing to ride in a self-driving car said it was due to concerns about safety and technology. The predominant sentiment was the perception that self-driving cars are not safe, and five respondents specifically noted they were aware of crashes involving self-driving cars. Multiple respondents said they would want to know more about the safety record of the vehicle before taking a ride, and would want more testing and “fine-tuning” of the vehicle to confirm it was a “proven” technology. A few respondents indicated they would want a larger volume of self-driving cars on the roadways before they felt they were safe to use.

Older adult trust of technology was also lacking. Multiple respondents said they “do not trust” self-driving cars, including a general distrust and lack of confidence in technology and computers. Respondents voiced a desire to have more control over the vehicle in certain situations—for example, to navigate traffic lights, stop signs, turns, and weather conditions. Others indicated they were fearful of the technology (respondents said: “*that gives me the creeps;” “I’m scared of those things;*” and “*it would take me a while to be comfortable in that situation*”). A few people commented they felt they were “too old” to ride in a self-driving car.

An additional reason respondents were not willing to use FAV ride sharing was the preference of having a driver. This was noted during discussion with 13 respondents, the majority of whom were users of ride share services. Many said they would not feel comfortable without a driver and would want someone reliable behind the wheel of the car. Three respondents said they wanted to have conversation and human interaction (one respondent said: “*I have so much fun with the drivers…the drivers are so smart and fun, so I would miss that in a driverless car*”). Two respondents specifically mentioned health concerns and said they would want a driver present to provide assistance in case of a medical issue (e.g., help out of the car due to vision impairment, assistance following a medical procedure).

Among those who would be willing to use FAV ride sharing, some older adults were enthusiastic (respondents said: “I would probably be excited”; “I would love to go down the road without a driver”; and “That would be unique; that would be enjoyable”). Other respondents indicated they would be willing to ride in a self-driving car as long as the technology worked and was “proven to be safe.”

Several of the respondents who were unsure about whether they would be willing use FAV ride sharing explained they did not know enough about the technology to make a decision or have an opinion on FAV ride sharing.

### 3.3. Themes Related to Desired Features of FAV Ride Sharing

There were four themes related to the desired features of self-driving cars that would make older adults willing to use FAV ride sharing: (1) a proven safety record; (2) dependability and accuracy; (3) ability to interface with the vehicle; and (4) ability to override the automated system. We discuss each theme below.

#### 3.3.1. Proven Safety Record

The strongest qualitative theme, noted by 31 older adult respondents, was the desire for a proven safety record in terms of vehicle performance and technology. This included additional testing of performance and technology, proof of overall functioning of the FAV, and FAV crash statistics. Sample quotes from respondents include: “*A safety record—they have not crashed*”; “*The fact that they had proven themselves completely reliable and safe*”; and “*Safety for me and as far as it being hacked or manipulated.*”

#### 3.3.2. Dependability and Accuracy

The theme of vehicle dependability and accuracy, noted by six respondents, was described in terms of transporting the rider to the correct location and ensuring a timely arrival at their destination. One respondent described dependability and accuracy as follows: “*How about that it delivers you to the appropriate drop off point. Meaning if it is the front door I need to go in, it’s not leaving me in a parking lot.*”

#### 3.3.3. Ability to Interface with the Vehicle

Some older adults wanted the ability to interface with the vehicle, such as through touch screens and audio notifications from a tablet or device. This theme was mentioned by five respondents. One respondent explained they would be willing to use FAV ride sharing, “*if I understood they were basically safe and I could use some kind of interface, a tablet, or something to run that. I would find that rather exciting.*” Some respondents were interested in receiving notifications about the location and arrival of the ride share service vehicle. For example, according to one respondent, “*There would need to be a feature that would give the customer the ability to identify the service when it’s there.*” Respondents did not indicate whether notifications from the service would be visual or auditory.

#### 3.3.4. Ability to Override the Automated System

A total of four respondents expressed a desire to have the ability to override the automated system. This theme was related to the desire to maintain some control over the vehicle, as explained by one respondent: “*I would like an override, yes. Although if it was that well engineered, there would be no need for an override because it would see and perceive any dangers far before I could perceive them.*”

## 4. Discussion

This paper provides new information regarding the promise of emerging self-driving car technology used by ride share services to serve as an alternative transportation option for older adults. The study explored older adult attitudes towards using FAV ride sharing, their willingness to do so, and the features of self-driving ride share vehicles that would make them willing to take a ride. The findings indicate that the majority of older adults (58.6%) were not willing to use FAV ride sharing, and less than one-quarter of older adults (21%) were willing to use FAV ride sharing. These findings are consistent with other studies that explored self-driving cars, such as West (2018), which found that 61% of older adults were not willing to ride in a self-driving car. Notably, very few respondents in this study were neutral or undecided about their willingness to use FAV ride sharing. Most older adults who were willing to try FAV ride sharing were enthusiastic about the prospect of doing so, and those who were not willing were strongly opposed.

Among the older adults who were not willing to use FAV ride sharing, many voiced strong concerns about the safety and reliability of the technology. They were fearful of and lacked trust in automated technology—findings that are consistent with other research in which older adults identified concerns about reliability, safety, and trust of self-driving cars [20,21]. Along with the fear and distrust noted by those who were not willing to use FAV ride sharing, the most important feature of a self-driving vehicle that would make older adults willing to take a ride was a proven safety record in terms of performance and technology. Indeed, existing research indicates that a low trust in technology is associated with negative attitudes toward self-driving vehicles [24] and that those with positive perceptions towards AV technology are more likely to be the early adopters [25]. Until the safety and reliability of FAVs can be fully demonstrated, it appears unlikely that older adults will be willing to adopt the technology and harness the benefits of FAV ride sharing as a transportation option. However, continued efforts to increase familiarity and exposure to FAV technology may help to shift negative perceptions among older adults, increasing their trust and perceived safety of the technology [5,17].

The study findings also revealed a preference, primarily among older adult users of ride share services, to have a human driver with them in the vehicle during a trip. In part, this feedback was in response to not feeling comfortable being in a car without a driver at the wheel. Other respondents noted a preference for having another individual in the vehicle to provide assistance, if needed, or social interaction and conversation. Research points to driver assistance and the opportunity for social interaction and conversation with drivers as facilitators of using ride share services among older adults [22], as is the opportunity to travel and socialize with friends [16]. Driver assistance may include a range of support, including door-through-door service, which is a preference of older adults who use ride share services and may be necessary for those with mobility challenges, health issues, or special needs [22]. In terms of social interaction, older adults who use non-profit ride share services report having built friendships with their drivers [22]. It is clear that human assistance and social connection are an integral and valued part of the ride share experience for older adults, and ride share services may need to account for these preferences when considering expanded use of FAVs, which eliminate the need for driver input. For-profit ride share services are exploring how to leverage FAVs, with the goal of removing the human driver to reduce costs and increase profitability [26]. However, ride share services may need to consider adapting their business models so that instead of drivers they offer mobility companions to older adults or people who need assistance. Future research could explore whether the availability of a mobility companion could affect the willingness of older adults to use FAV ride sharing and whether the role of volunteer drivers for non-profit ride share services could evolve from driver to companion.

Knoefel and colleagues (2019) noted that AV availability could profoundly affect the mobility of older adults who can no longer perform the tasks required for safe driving due to cognitive impairments: “In a similar way that glasses aid in visual clarity, and walkers support walking independence, driving automation could help decrease the impact of cognitive change on older adult driving” [6]. While ride sharing is a transportation option that stands to mitigate the consequences of driving cessation and support continued mobility of older adults, many services including TNCs are only in the beginning stages of creating systems to address the needs of people with special mobility considerations [4]. Considering the needs and preferences of older adults, such as the preference for social interaction and driver assistance, is crucial for ensuring that transportation options available to older adults can support their mobility and independence. As ride share services consider implementing FAV technology for use by older adults, it is also essential to consider their concerns about safety and lack of trust in technology and acknowledge that due to these concerns, older adults are unlikely to be early adopters of this technology. However, as noted, increased exposure to AV technology may increase older adult comfort with self-driving vehicles [5,17]. There are also opportunities to increase training on the technology so that older adults better understand AV technology and its usage [6,19]. Identifying opportunities to involve older adults in AVs, over time, may increase comfort and willingness to use FAV ride sharing among older adults.

### Limitations

The study has the following potential limitations, which should be considered. First, it is unknown to what extent study participants were familiar with or knowledgeable about the different levels of driving automation and self-driving cars in particular. Therefore, there is the potential for confusion regarding the definition of self-driving or “driverless” automobiles, which are fully automated, and other levels of automation. Future studies can take into account familiarity with self-driving vehicles when recruiting participants. Second, due to the fluid nature of focus group discussions, and despite encouraging open dialogue, not all study participants provided a response to the question about driverless cars. Third, study participants were recruited through convenience sampling and thus the results may not represent the general population of older adults in the U.S. Fourth, the views of racially and ethnically diverse respondents were not adequately reflected, as the majority of study participants were white and not Hispanic or Latino. Future studies can seek to achieve both a more representative and more diverse sample of older adults. Finally, the ability to explore whether contextual or demographic factors were correlated with willingness to use FAV ride sharing was limited by the study design. Future research may seek to further investigate these factors.

## 5. Conclusions

Ride sharing has the potential to support older adult mobility and independence, and FAV ride sharing is an emerging technology that shows promise as an alternative transportation option for older adults in the future. In this study, the majority of older adults indicated that they were not willing to use FAV ride sharing, citing concerns about safety and trust of technology. As FAVs enter the market, there remain opportunities for increasing acceptance of the technology among older adults. In addition to a proven safety record, other features of FAV ride sharing that may increase acceptance among older adults include dependability and ability to interact with the vehicle. However, FAV ride sharing may not be able to replace the social experience that many older adults want and need during their trips, and in the future, ride share services may need to consider how to adapt their business models to account for older adult preferences for companionship. These insights may help ride share and other transportation services understand the needs and preferences of older adults as they consider strategies for supporting their continued mobility and independence.

## Figures and Tables

**Table 1:** Characteristics of older adult participants, Barriers and Facilitators of Older Adults’ Use of Ride Share Services Study, 2019, *n* = 124 ^a^.

Characteristic		*n*	%
Age Group ^b^			
	65–74	62	52.5%
	75–84	32	27.1%
	85+	24	20.3%
Gender			
	Female	87	70.2%
	Male	37	29.8%
Geographic Location			
	Large metro area (1+ million residents)	78	63.9%
	Small metro area (<1 million residents) ^c^	44	36.1%
Race			
	White	116	97.5%
	Black	2	1.7%
	Asian	1	0.8%
Hispanic or Latino			
	No	121	98.4%
	Yes	2	1.6%
Use of ride share services			
	No, never used a ride share service	29	23.4%
	Yes, for-profit service	41	33.1%
	Yes, non-profit services	38	30.6%
	Yes, both for-profit and non-profit services	15	12.1%
Driver’s license			
	Yes	88	72.1%
	No	34	27.9%

^a^ Counts and calculated percentages account for missing data due to nonresponse; therefore, values may not sum to the total number of study respondents (*n* = 124). ^b^ Age group excludes six (6) respondents who were age 65+ but did not provide their exact age or age group. ^c^ Includes one respondent from a non-metropolitan community.

**Table 2:** Willingness of older adult participants to use fully autonomous vehicle ride sharing, Barriers and Facilitators of Older Adults’ Use of Ride Share Services Study, 2019, *n* = 124.

	*n*	%
No, not willing	73	58.9%
Yes, willing	26	21.0%
Unsure	7	5.7%
No response	18	14.5%
Total	124	100.0%

## Data Availability

The data gathered in this study did not result in a dataset available for public release and have been archived in accordance to contract terms.
